# Potential bias in the clinical trial registration of patients with peripheral small-sized non-small cell lung cancer: Real-world data in the era of segmentectomy

**DOI:** 10.1007/s00595-026-03259-3

**Published:** 2026-03-03

**Authors:** Yuji Nomata, Shota Nakamura, Keita Nakanishi, Yuka Kadomatsu, Harushi Ueno, Taketo Kato, Tetsuya Mizuno, Toyofumi Fengshi Chen-Yoshikawa

**Affiliations:** Department of Thoracic Surgery, Nagoya University Graduate School of Medicine, 65 Tsurumai-cho, Showa-ku, Nagoya, 466-8550 Japan

**Keywords:** Non-small-cell lung cancer, Early-stage peripheral lung cancer, Sublobar resection, Selection bias

## Abstract

**Purpose:**

Recent phase III trials demonstrate that survival after segmentectomy is not inferior to that after lobectomy for peripheral small-sized non-small cell lung cancer (NSCLC). However, the influence of patient selection and registration bias remains unclear. This study analyzed real-world data to assess patients who were eligible for the JCOG0802/WJOG4607L trial, but who were not enrolled.

**Methods:**

We analyzed data on 38 of 61 patients not enrolled in the JCOG0802/WJOG4607L trial, during its enrollment period. Surgical procedures, clinical characteristics, and outcomes of the nonregistered patients were reviewed.

**Results:**

Lobectomy was performed in 31 patients and segmentectomy was performed in 7 patients. Radiological features of higher-grade malignancy were seen more frequently in the patients who underwent lobectomy. The most common reason for nonregistration in the JCOG0802/WJOG4607L trial was multiple mild comorbidities (*n* = 11), followed by tumor aggressiveness (*n* = 6), and concerns about inadequate margins with segmentectomy (*n* = 6). Recurrence was found in five patients. The 5-year overall and recurrence-free survival rates were 82.9% and 80.2%, respectively.

**Conclusions:**

Our analysis emphasizes the challenges posed by registration bias in clinical trials. Survival outcomes among the study population were worse than those reported in the JCOG0802/WJOG4607L trial, suggesting a high malignant potential on preoperative imaging, and that registration bias occurs even in large-scale randomized trials.

## Introduction

Pulmonary lobectomy has been the standard surgical procedure for lung cancer treatment for several decades and is recognized widely as the most effective approach for achieving local control [1, 2]. The introduction and development of various minimally invasive methods, such as video-assisted thoracoscopic surgery and robot-assisted thoracic surgery, have increased the number of sublobar resections being performed as a lung-sparing minimal invasive surgical procedure [3, 4]. Several studies over the past two decades have revealed comparable prognoses for small, early-stage peripheral lung cancers treated with sublobar resections [5]. Based on these results, two large-scale, multi-institutional phase III trials were conducted to identify the optimal surgical approach for patients with small peripheral non-small cell lung cancer (NSCLC) [6, 7]. These trials revealed the noninferiority of sublobar resection to lobectomy in terms of overall survival (OS) and recurrence-free survival (RFS).

Results from prospective randomized phase III trials revealed high-level scientific evidence and played a pivotal role in shaping standard treatments [8–10]. However, the decision to participate depends on the will of each patient, even for those who meet the eligibility criteria for such trials. Furthermore, treating physicians, who provide explanations concerning the disease and the clinical trial, frequently hold personal beliefs or biases regarding the treatment options or trial itself. These predispositions may affect the information conveyed to patients and subsequently influence their decision-making [11, 12].

Our institution participated in the JCOG0802/WJOG4607L trial, led by the Japan Clinical Oncology Group and the West Japan Oncology Group, which compared lobectomy and segmentectomy for small peripheral NSCLC. However, not all eligible patients at our institution were enrolled in the trial. Various factors contributed to this nonregistration, and potential biases in patient registration may have affected the outcomes and consequently, the overall results of the trial. To address this issue, we analyzed eligible patients from our institution, who were not enrolled in the trial, by investigating their demographics, pathological findings, surgical procedures, and clinical outcomes. This study aimed to investigate the potential biases that appeared even in phase III randomized trials, and to explore the real-world data and implications of these biases.

## Materials and methods

### Ethical consideration

All studies that involved human participants were conducted under the ethical standards outlined by institutional and national research committees and the 1964 Declaration of Helsinki, along with its subsequent amendments or equivalent ethical standards. This retrospective study obtained authorization from the review boards of Nagoya University Hospital (No. 2017-0034).

### Patients

**Fig. 1:**
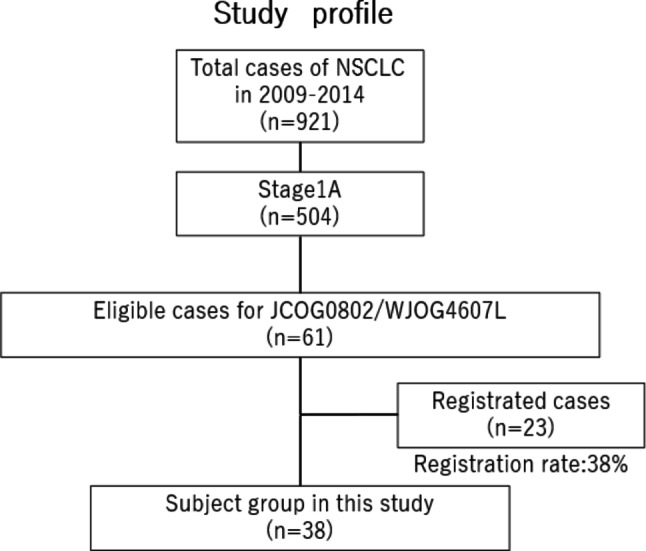
Study flow diagram of eligible and non-registered patients during the JCOG0802/WJOG4607L trial period. A total of 61 cases were eligible with criteria in the trial of JCOG 0802. In those patients, 38 eligible cases were not registered in the trial and were included in this analysis

We collected patient data from Nagoya University Hospital, focusing on patients diagnosed with peripheral small-sized NSCLC from October, 2009 to August, 2014. During this period, the randomized controlled trial JCOG0802/WJOG4607L was ongoing in Japan, and eligible patients were enrolled in accordance with the trial’s criteria. In total, 61 patients met the eligibility criteria, but 38 of these patients were not registered in the trial and thus, were included in this analysis (Fig. 1).

The eligibility criteria for this analysis as well as for JCOG0802/WJOG4607L are outlined below.

#### Tumor characteristic criteria

1. Single, primary, small-sized, invasive peripheral NSCLC (diameter of ≤ 2 cm; consolidation-to-tumor (C/T) ratio of > 0·5; location in the outer one-third of the pulmonary parenchyma) confirmed through thin-section preoperative, contrast-enhanced thoracic computed tomography (CT).
2. The tumor was not located in the middle lobe and there was no evidence of lymph node metastasis.

#### Patient characteristic criteria

1. Eligible patients aged 20–85 years with an Eastern Cooperative Oncology Group performance score (ECOG–PS) of 0 or 1.
2. No history of ipsilateral thoracotomy or previous chemotherapy or radiotherapy for any malignant diseases.
3. An expected postoperative forced expiratory volume in 1 s (FEV1) of ≥ 800 mL and a partial pressure of arterial oxygen of ≥ 65 mmHg.
4. Sufficient organ function.
5. Written informed consent for enrollment was provided.

#### Exclusion criteria

Patients who met any of the following criteria were excluded from the trial:

1. Active bacterial or fungal infection.
2. Simultaneous or metachronous (within the past 5 years) double cancers.
3. Women during pregnancy or breastfeeding.
4. Interstitial pneumonitis, pulmonary fibrosis, or severe pulmonary emphysema.
5. Psychosis.
6. Systemic steroidal medication.
7. Uncontrollable diabetes.
8. Uncontrollable hypertension.
9. A history of severe heart disease.

### Database and patient follow-up

The following clinical and pathological characteristics were recorded in our database: age, sex, smoking history, Charlson comorbidity index, ECOG–PS, FEV1, forced vital capacity, tumor size, C/T ratio, maximum standardized uptake value (SUVmax), preoperative carcinoembryonic antigen levels, pathological type, and pathological stage based on the seventh edition of the American Joint Committee on Cancer Tumor–Node–Metastasis (AJCC-TNM) classification. The tumor was diagnosed pathologically using the World Health Organization classification of tumors. All characteristics were recorded according to surgical procedure type.

Patients were followed up in an outpatient clinic at 3-month intervals for the first 2 years postoperatively and at 6-month intervals thereafter. Patients receiving postoperative adjuvant therapy were monitored by blood tests during this period. Recurrence or survival data were collected, whenever possible, for up to 10 years. Recurrence was diagnosed based on compatible physical examination results and/or diagnostic imaging, with histological confirmation when clinically feasible.

### Definition of OS and RFS

OS was defined as the interval between the surgery date and death from any cause, or the last follow-up. RFS was defined as the interval between the date of surgery and the date of recurrence detection, death from any cause, or the last follow-up. Observations were censored at the final follow-up when the patient was alive or lost to follow-up. February 2024 was the data cutoff date.

### Statistical analysis

Categorical variables included age (< 75 or ≥ 75 years), sex, smoking history, comorbidity score (Charlson comorbidity index), surgical procedure type, pathological type, and pathological stage based on the eighth edition of the TNM classification. The Kaplan–Meier method was used for survival curve estimation, and the log-rank test was used to compare differences in OS and RFS. Data were analyzed using the IBM Statistical Package for the Social Sciences Statistics (version 29.0.1.0).

## Results

**Fig. 2:**
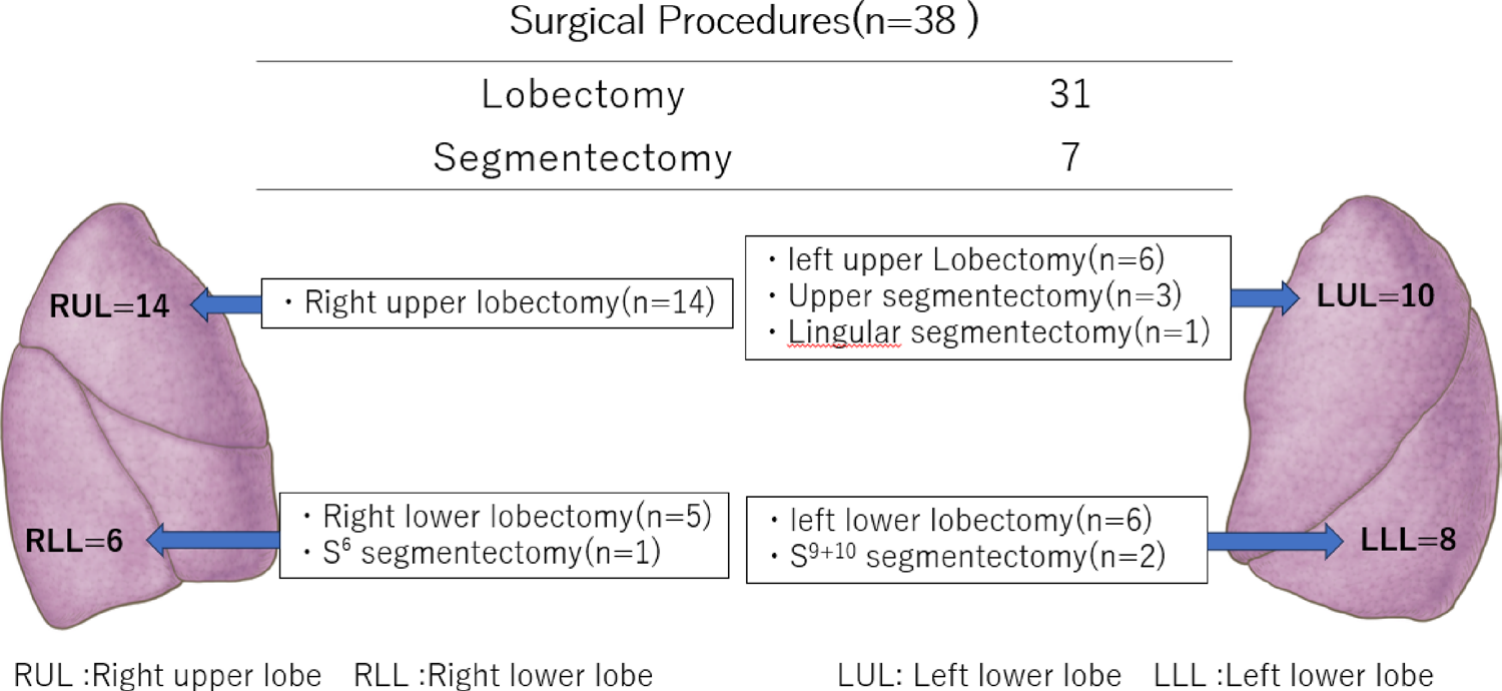
Distribution of surgical procedures according to tumor location (*n* = 38). Number of patients who underwent lobectomy or segmentectomy in each lung lobe and the corresponding surgical procedures performed

Table 1 summarizes the clinical characteristics of the 38 nonregistered patients, including demographic data, tumor-related imaging findings (C/T ratio and SUVmax), and pulmonary function. Data were further stratified based on surgical procedure (lobectomy vs. segmentectomy), highlighting differences in baseline factors. Figure 2 illustrates the anatomical distribution of tumors and the corresponding surgical procedures performed in each patient. Those who underwent segmentectomy were emphasized to indicate the rationale for their selection. Thirty-one (81.6%) of the 38 patients underwent pulmonary lobectomy and 7 (18.4%) underwent segmentectomy. Patients who underwent lobectomy had a higher C/T ratio and SUVmax value of the nodule (median: 1.0) than those who underwent segmentectomy (Table 1). Twenty of the 31 patients who underwent lobectomy had pathologically confirmed stage IA disease, consistent with their preoperative diagnosis. Six patients were found to have more advanced stage disease than estimated from preoperative imaging. Conversely, patients who underwent segmentectomy had lower respiratory function and SUVmax values than those who underwent lobectomy. Six of the seven patients who underwent segmentectomy had pathologically confirmed stage IA disease, whereas one had more advanced disease than initially expected.

**Table 1:** Clinical characteristics of the non-registered patients, stratified by surgical procedure (lobectomy vs. segmentectomy)

Patient characteristics	Overall enrolled patients	Patients who underwent lobectomy	Patients who underwent segmentectomy
	*n* = 38	*n* = 31	*n* = 7
Age (years)			
Median (range)	67 (45–82)	65 (26–74)	77 (43–91)
*Sex*			
Female	14(36.8%)	9(29.0%)	5(71.4%)
Male	24 (63.2%)	22(71.0%)	2 (28.6%)
*Smoking history*			
Never	15(39.5%)	10 (32.3%)	5 (71.4%)
Experienced/Current	23 (60.5%)	21(67.7%)	2(28.6%)
ECOG-PS* 0/1(%)			
0	23(60.5%)	20(64.5%)	3(42.9%)
1	15(39.5%)	11(35.5%)	4(57.1%)
*Charlson comorbidity index*			
Median(range)	4(1–9)	4(1–9)	5(3–8)
FVC(ml)			
Median (range)	3280 (1780-4700)	3460 (1780-4700)	2700 (2010-3020)
*FEV1(ml)*			
Median (range)	2360 (1200-3920)	2550 (1200-3920)	1730 (1370-2100)
Tumor size(cm)			
Median(range)	1.6(0.8–2.0.8.0)	1.7(0.8–2.0.8.0)	1.4(1.1–1.9.1.9)
Pleural indentation on CT image			
Present	21(55.3%)	17(54.8%)	4(57.1%)
Absent	17(44.7%)	14(45.2%)	3(42.9%)
C/T ratio**			
Median(range)	0.9(0.51–1.0.51.0)	1.0(0.51–1.0.51.0)	0.8(0.71–1.0.71.0)
SUVmax***			
Median(range)	3.9(1.3–13.3.3.3)	4.0(1.3–13.3.3.3)	1.9(1.6–6.8.6.8)
CEA****			
Median(range)	2.2(1.0–8.6.0.6)	2.2(1.0–8.6.0.6)	2.1(1.2–5.4.2.4)
Histopathology			
Adenocarcinoma	29(76.3%)	23(74.2%)	6(85.7%)
Squamous cell Carcinoma	7(18.4%)	6(19.3%)	1(14.3%)
Adenosquamous Carcinoma	2(5.3%)	2(6.5%)	0(0.0%)
*Pathological Stage*			
T1aN0 T1bN0 T2aN0 T2aN1 T1aN2 T1bN2	24(63.1%) 2(5.3%) 8(21.1%) 1(2.6%) 1(2.6%) 2(5.3%)	19(61.3%) 1(3.2%) 7(22.6%) 1(3.2%) 1(3.2%) 2(6.5%)	5(71.4%) 1(14.3%) 1(14.3%) 0(0.0%) 0(0.0%) 0(0.0%)

* ECOG-PS: Eastern Cooperative Oncology Group Performance Status

* * C/T ratio: C/T ratio: Consolidation-to-Tumor Rati

*** SUVmax: Maximum Standardized Uptake Value

**** CEA: Carcinoembryonic Antigen

### Postoperative treatment

One of the 12 patients who was found to have more advanced pathological stage disease than initially thought, based on preoperative imaging, received adjuvant chemotherapy with tegafur–uracil (UFT) for stage IB disease. Three others with pathological N2 disease received postoperative cisplatin-based doublet chemotherapy. The remaining seven patients did not receive adjuvant therapy because of advanced age or their refusal. None of the segmentectomy patients with advanced pathological stage received postoperative adjuvant treatment.

### Reasons for nonregistration

**Fig. 3:**
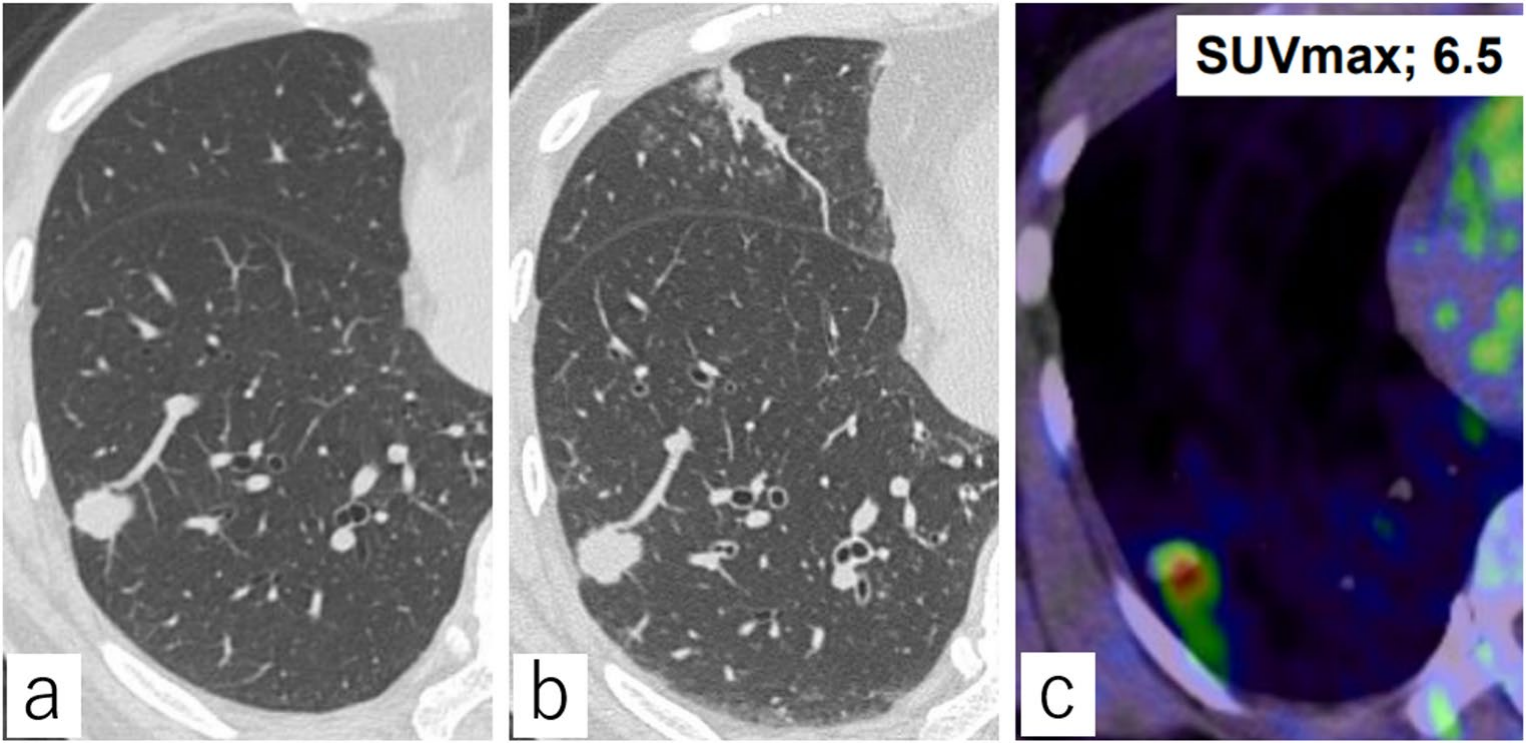
Representative case with radiological features suggesting high tumor aggressiveness. (**a**) A 48-year-old man with a 1.7-cm pure solid tumor located in the right S9 at initial presentation. (**b**) The tumor demonstrated rapid growth, increasing to 2.0 cm within one month. (**c**) Fluorodeoxyglucose (FDG) positron emission tomography showed high uptake (SUVmax, 6.5). He underwent a right lower lobectomy and has remained free from recurrence for 124 months

The most common reason for non-registration of the 38 patients who were eligible for the JCOG0802/WJOG4607L trial but were not registered, was the co-existence of multiple mild comorbidities (*n* = 11). In six patients, preoperative CT and positron emission tomography (PET) imaging suggested high tumor aggressiveness. Accordingly, the surgical team recommended lobectomy as the standard approach. For example, preoperative CT imaging in a 48-year-old man revealed a 1.7-cm pure-solid tumor in the right lower lobe of S^9^ (Fig. 3a, b) and PET/CT revealed intense fluorodeoxyglucose (FDG) uptake (SUVmax: 6.5) corresponding to the tumor shadow (Fig. 3c). Moreover, the tumor demonstrated a remarkably rapid growth rate over a short period, further supporting the decision to perform lobectomy rather than segmentectomy. At the time of writing, 124 months after the lobectomy, the patient is alive without any sign of recurrence. Six patients who underwent lobectomy were not registered in the clinical trial because it was thought that segmentectomy has a shorter surgical margin.

**Fig. 4:**
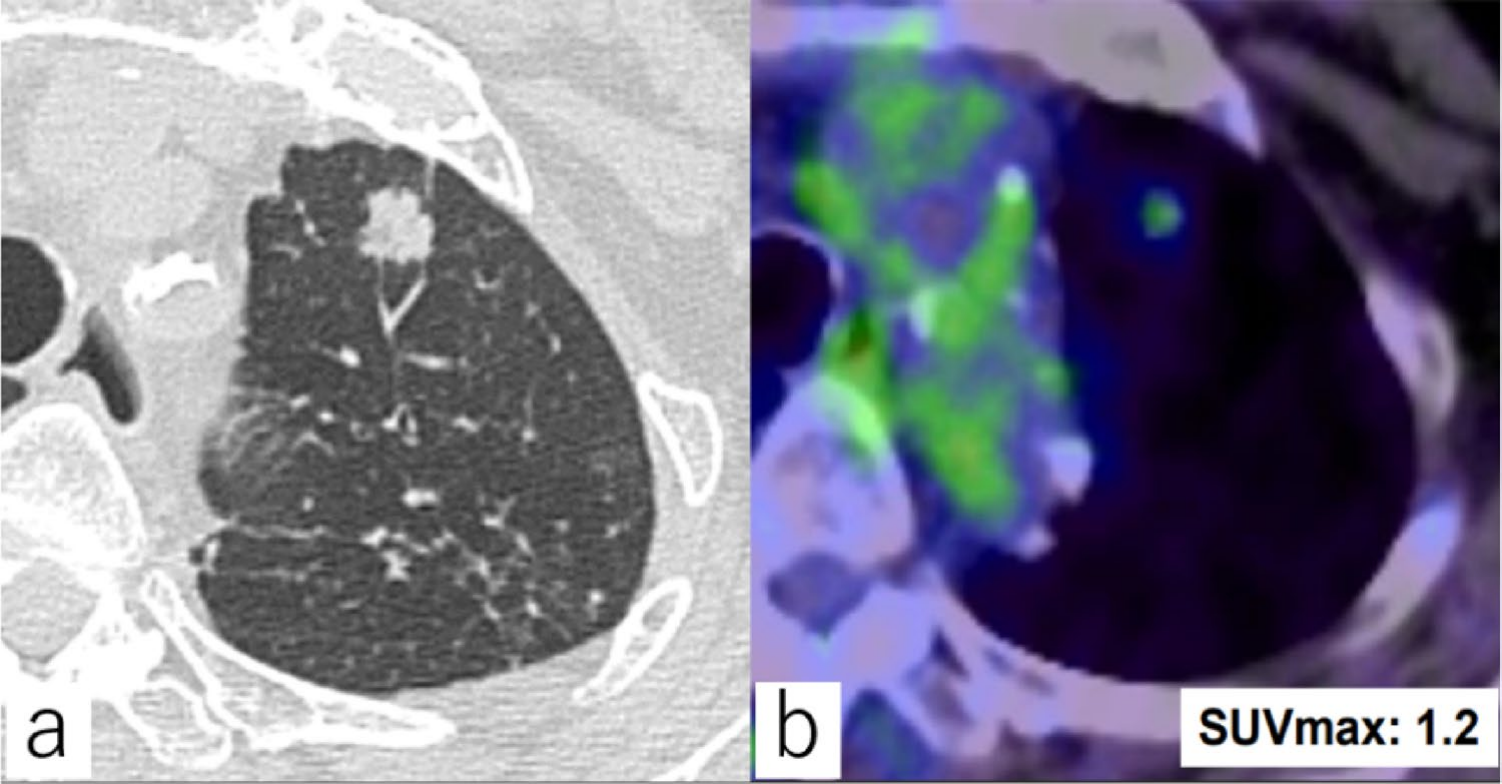
Representative case with radiological features suggesting low tumor aggressiveness. (**a**) A 65-year-old woman, diagnosed with adenocarcinoma, presenting with a 1.4-cm tumor in the left S1+2 tumor, with a consolidation-to-tumor (C/T) ratio of 0.71. (**b**) The tumor showed low FDG uptake (SUVmax, 1.2). She underwent a left superior segmentectomy and has remained recurrence-free for 37 months after surgery

Two patients who underwent segmentectomy were not registered in the clinical trial because CT and PET imaging showed low biological aggressiveness of the tumor. Segmentectomy was deemed sufficient to achieve a safe resection margin, thereby ensuring an adequate preventive effect against recurrence for those patients. CT imaging in a 65-year-old woman revealed a 1.4-cm part-solid nodule in the left upper lobe of S^1 + 2^, which was diagnosed as lung adenocarcinoma (Fig. 4a). The C/T ratio was 0.71, and PET imaging revealed no significant FDG uptake in the tumor, with an SUVmax of 1.2 (Fig. 4b). Segmentectomy was expected to achieve a sufficient surgical margin; thus, intentional left superior segmentectomy was performed. At the time of writing, 37 months after the segmentectomy, the patient is alive without any sign of recurrence. Five patients declined to register in the clinical trial. Among the remaining nine patients who were not registered, four opted for postoperative follow-up at another hospital nearer their home, one resided abroad, and the reasons for non-enrolment of four patients were unknown.

### Patients with recurrence

Recurrence developed during the follow up period in 5 of the 38 patients. Table 2 summarizes the characteristics of the five patients with recurrence. One of these five had undergone segmentectomy. In one patient with an epidermal growth factor receptor (EGFR) mutation, treatment with an EGFR-tyrosine kinase inhibitor was initiated after recurrence, which resulted in survival for more than 3 years post-recurrence. The remaining four patients died of the disease within 1–14 months post-recurrence. The median postoperative survival time for the five patients with recurrence was 51 months, and all patients died within 10 years of surgery.

**Table 2:** Characteristics of the patients with recurrence (*n*=5)

	Procedure	Histopathology	Pathological Stage	Adjuvant therapy	Site of recurrence	Chemotherapy for recurrence	Post-operative survival	post- recurrence survival	Status
Patient 1	Right upper lobectomy	Squamous call carcinoma	T1aN0	None	Lymph nodes+ Pulmonary metastasis	Carboplatin+S-1	58 months	1 month	Died of disease
Patient 2	Right upper lobectomy	Adenocarcinoma	T2aN0	None	Bronchus +Bone	EGFR-tyrosine kinase inhibitor	75 months	43 months	Died of disease
Patient 3	Left upper lobectomy	Squamous call carcinoma	T2aN1	None	Dissemination	Carboplatin+S-1	38 months	14 months	Died of disease
Patient 4	Left S^9+10^ Segmentectomy	Squamous call carcinoma	T1bN0	None	Pulmonary metastasis	None	51 months	2 months	Died of disease
Patient 5	Right upper lobectomy	Adenocarcinoma	T1aN0	None	Lymph nodes	None	12 months	3 months	Died of disease

EGFR, Epidermal Growth Factor Receptor

### OS and RFS

**Fig. 5:**
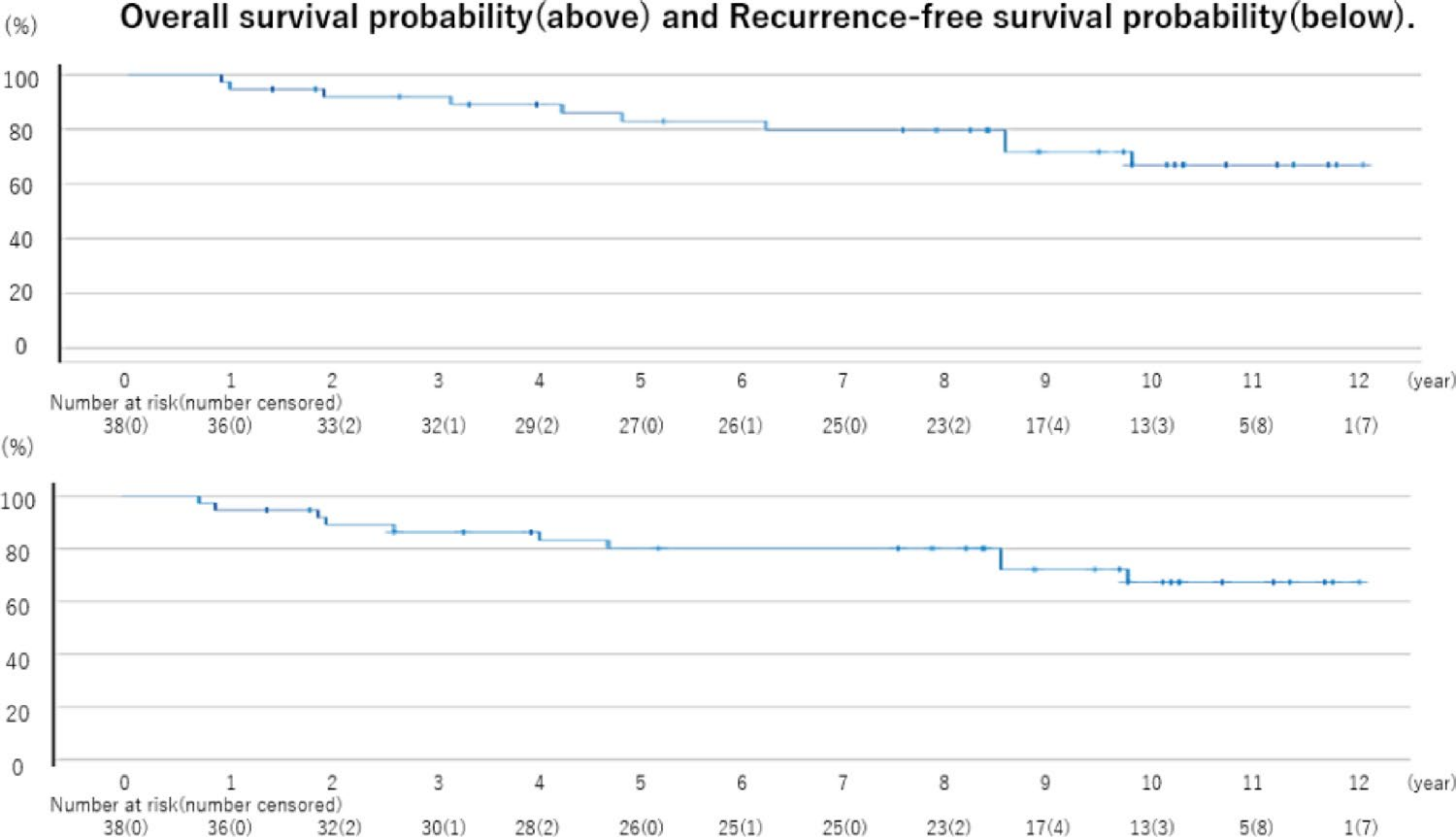
Overall and recurrence-free survival in trial-eligible but non-registered patients. Overall survival probability(above) and Recurrence-free survival probability(below)

Figure 5 shows the OS and RFS of all the enrolled patients. The median follow-up time was 103 months. The 5-year OS and RFS rates were 82.9% and 80.2%, and the 10-year OS and RFS rates were 67.0% and 67.4%, respectively.

## Discussion

For several decades, lobectomy has been the standard treatment for peripheral small-sized NSCLC, supported by evidence from phase III trials that indicated its superiority over limited resection in terms of survival and local control [2]. Advances in imaging technology have enabled the detection of less invasive lung cancers [13], which has prompted a shift in practice toward sublobar resections for selected cases, with favorable outcomes [5, 14–16]. Recent trials that reported sublobar resections, including segmentectomy and wedge resection, which were not inferior to lobectomy, have further confirmed these paradigm shifts in surgical procedures [17]. A phase III trial comparing lobectomy and segmentectomy for patients with small peripheral NSCLC revealed that segmentectomy, when adequate surgical margins are achieved, was comparable to lobectomy in terms of OS and RFS [6]. Twenty-three patients from our institution were registered in this trial, contributing to the overall accrual; however, during the accrual period, 61 eligible patients were identified at our facility, with 38 not enrolled. Recognizing that registration bias could affect trial outcomes, we conducted the present study to investigate the characteristics and clinical outcomes of the eligible but nonregistered patients. This allowed us to identify patterns in surgical decision-making that emphasized how clinical judgment and patient-specific factors influence trial participation, aiming to provide a unique perspective on the challenges of implementing evidence from controlled trials into daily practice.

Among the 38 nonregistered patients, 6 with high-grade malignant tumors on image findings underwent lobectomy, and another 6 without sufficient margins on segmentectomy required lobectomy. Conversely, two patients underwent segmentectomy because of low malignancy potential inferred from imaging. Comorbidities, although within the trial eligibility criteria, were a significant factor that affected registration. This study did not enroll 11 patients who were eligible but had multiple minor complications, after consultation with the patients, as a limited procedure was considered preferable. Thus, for 25 of the 38 patients, the surgeon’s indications may have affected the patient’s decision, resulting in non-enrolment despite eligibility for the trial. Similar situations likely occur at other participating institutions.

The patients enrolled in the present study may have been eligible for enrollment in the JCOG0802/WJOG4607L trial, considering that in the trial, the 5-year OS was 94.3% and 91.1% and the 5-year RFS was 88.0% and 87.9% for the segmentectomy and lobectomy groups, respectively. However, the patients included in the present study had poorer oncological outcomes. These facts indicate that surgeons’ clinical judgments, including recommendations or opinions about tumors suspected to have high malignant potential, as well as patient-specific factors, may affect decisions about trial participation. This study reflects the experience of a single institution and does not provide definitive evidence; however, it suggests the potential presence of selection bias that may affect trial enrollment patterns.

In clinical trials, selection bias occurs while excluding patients with a poor prognosis or those with favorable outcomes and a clear treatment advantage. Such biases frequently arise from physicians’ clinical judgment and the prioritization of individual patient care over trial participation. According to Rothwell, the impact of this bias is minimal when the tested treatment has a clear advantage over the control. However, when the treatment’s superiority is uncertain, the bias may threaten the study’s external validity [18]. Registration bias may not be eliminated even in large-scale, highly reliable prospective randomized controlled trials if the superiority of the treatment being tested is uncertain. This bias has been reported in randomized controlled trials (RCTs) across oncology as well as other disease areas. Identifying the characteristics of nonregistered patients, as in the present study, represents one approach to evaluating the external validity of trial findings. Recognizing its existence in clinical practice allows for a more nuanced interpretation of RCT results. For example, when using the results of a given RCT to guide patient selection or surgical strategy, clinicians can consider the possibility that certain high- or low-risk patient groups may have been excluded. This provides important context for assessing whether reported survival or recurrence rates are applicable to their own patient population. Thus, awareness of registration bias can facilitate more individualized and appropriate treatment decisions in routine clinical practice.

The CSPOR-LC03 trial, conducted in relation to the JCOG0707 trial, indicates the presence of these biases. The JCOG0707 trial was a phase III randomized controlled trial that compared the efficacy of UFT and tegafur–gimeraci–oteracil (S-1) in patients with stage I NSCLC, who had undergone complete resection [19]. The CSPOR-LC03 trial focused on patients who were eligible for JCOG0707 but were not registered. The CSPOR-LC03 trial emphasizes that even in large-scale phase III trials, registration bias affects results significantly, as healthier patients may be registered and those with poor prognoses may be excluded based on subjective clinical judgment [20]. Although a direct comparison is not possible, our results resonate with the conceptual framework emphasized in the CSPOR-LC03 analysis. This perspective emphasizes the importance of accounting for registration patterns when evaluating the generalizability of randomized trial outcomes, particularly in real-world clinical practice.

The 38 patients who were eligible for the study but were not enrolled suggest registration bias; however, this does not imply the superiority of either lobectomy or segmentectomy, and further studies are needed to clarify the role of segmentectomy. Interestingly, four of the five cases of recurrence in our cohort occurred in the lobectomy group, suggesting that the choice of surgical procedure may not always impact prognosis significantly. This finding does not contradict the results of the JCOG0802/WJOG4607L trial. In fact, our study aims to complement the results of the JCOG0802/WJOG4607L trial by demonstrating the existence of registration bias and highlighting the effects of real-world clinical decision-making on trial enrollment patterns.

The limitation of this study was the small number of patients analyzed. The limited number of segmentectomies (*n* = 7) made it impossible to compare survival outcomes between surgical procedures. This study is hypothesis-generating and not designed to evaluate the efficacy of a specific surgical procedure. Collaboration with other institutions, ideally involving all facilities participating in phase III trials, and the use of multicenter or registry-based studies could provide a larger and more diverse dataset, enabling more robust comparisons and deeper insights into the association between registration bias and trial outcomes. Future studies addressing these biases may help refine trial methodologies and improve the applicability of their results to real-world settings.

## Conclusion

The current analysis of patients who were eligible for but not registered in a clinical trial reveals the challenges posed by registration bias in clinical trials. The survival outcomes of these patients were worse than those reported in the JCOG0802/WJOG4607L trial, likely indicating a higher malignant potential on preoperative imaging. These results indicate that registration bias occurs even in large-scale randomized trials.

## References

[1] Cahan WG. Radical lobectomy. J Thorac Cardiovasc Surg. 1960;39:555–72. 13806783

[2] Ginsberg RJ, Rubinstein LV. Randomized trial of lobectomy versus limited resection for T1 N0 non-small cell lung cancer. Lung Cancer Study Group. Ann Thorac Surg. 1995;60:615–22. discussion 622–623. 7677489 10.1016/0003-4975(95)00537-u

[3] Chen-Yoshikawa TF, Fukui T, Nakamura S, Ito T, Kadomatsu Y, Tsubouchi H, et al. Current Trends in Thoracic Surgery. Nagoya J Med Sci. 2020;82(2):161–74. 32581397 10.18999/nagjms.82.2.161 PMC7276403

[4] Ueno H, Imamura Y, Okado S, Nomata Y, Watanabe H, Kawasumi Y, et al. Lobectomy for primary lung cancer: a comparison of perioperative and postoperative outcomes between robot-assisted thoracic surgery and video-assisted thoracic surgery. Surg Today. 2025;55(8):1162–72. 39960546 10.1007/s00595-025-03000-6 PMC12339585

[5] Okada M, Koike T, Higashiyama M, Yamato Y, Kodama K, Tsubota N. Radical sublobar resection for small-sized non-small cell lung cancer: a multicenter study. J Thorac Cardiovasc Surg. 2006;132(4):769–75. 17000286 10.1016/j.jtcvs.2006.02.063

[6] Saji H, Okada M, Tsuboi M, Nakajima R, Suzuki K, Aokage K, et al. Segmentectomy versus lobectomy in small-sized peripheral non-small-cell lung cancer (JCOG0802/WJOG4607L): a multicentre, open-label, phase 3, randomised, controlled, non-inferiority trial. Lancet. 2022;399(10335):1607–17. 10.1016/S0140-6736(22)00446-2. 35461558 10.1016/S0140-6736(21)02333-3

[7] Altorki N, Wang X, Kozono D, Watt C, Landrenau R, Wigle D, et al. Lobar or sublobar resection for peripheral stage IA non-small-cell lung cancer. N Engl J Med. 2023;388(6):489–98. 36780674 10.1056/NEJMoa2212083 PMC10036605

[8] Concato J, Shah N, Horwitz RI. Randomized, controlled trials, observational studies, and the hierarchy of research designs. N Engl J Med. 2000;342(25):1887–92. 10861325 10.1056/NEJM200006223422507 PMC1557642

[9] Sackett DL, Rosenberg WM, Gray JA, Haynes RB, Richardson WS. Evidence based medicine: what it is and what it isn’t. BMJ. 1996;312(7023):71–2. 8555924 10.1136/bmj.312.7023.71 PMC2349778

[10] Ioannidis JPA. Why most clinical research is not useful. PLoS Med. 2016;13(6):e1002049. 27328301 10.1371/journal.pmed.1002049 PMC4915619

[11] Joffe S, Cook EF, Cleary PD, Clark JW, Weeks JC. Quality of informed consent: a new measure of understanding among research subjects. J Natl Cancer Inst. 2001;93(2):139–47. 11208884 10.1093/jnci/93.2.139

[12] Albrecht TL, Blanchard C, Ruckdeschel JC, Coovert M, Strongbow R. Strategic physician communication and oncology clinical trials. J Clin Oncol. 1999;17(10):3324–32. 10506636 10.1200/JCO.1999.17.10.3324

[13] Suzuki K, Koike T, Asakawa T, Kusumoto M, Asamura H, Nagai K, et al. A prospective radiological study of thin-section computed tomography to predict pathological non-invasiveness in peripheral clinical IA lung cancer (JCOG0201). J Thorac Oncol. 2011;6(6):751–6. 21325976 10.1097/JTO.0b013e31821038ab

[14] Yamato Y, Tsuchida M, Watanabe T, Aoki T, Koizumi N, Umezu H, et al. Early results of a prospective study of limited resection for bronchioloalveolar adenocarcinoma of the lung. Ann Thorac Surg. 2001;71:971–4. 11269483 10.1016/s0003-4975(00)02507-8

[15] Tsubota N, Ayabe K, Doi O, Mori T, Namikawa S, Taki T, et al. Ongoing prospective study of segmentectomy for small lung tumors. Study Group of Extended Segmentectomy for Small Lung Tumor. Ann Thorac Surg. 1998;66:1787–90. 9875790 10.1016/s0003-4975(98)00819-4

[16] Kodama K, Doi O, Higashiyama M, Yokouchi H. Intentional limited resection for selected patients with T1 N0 M0 non-small-cell lung cancer: a single-institution study. J Thorac Cardiovasc Surg. 1997;114:347–53. 9305186 10.1016/S0022-5223(97)70179-X

[17] Suzuki K, Watanabe S, Wakabayashi M, Moriya Y, Yoshino I, Tsuboi M, et al. A nonrandomized confirmatory phase III study of sublobar surgical resection for peripheral ground glass opacity dominant lung cancer defined with thoracic thin-section computed tomography (JCOG0804/WJOG4507L). J Clin Oncol. 2017;35(Suppl):abstr8561.

[18] Rothwell PM. External validity of randomised controlled trials: to whom do the results of this trial apply? Lancet. 2005;365(9453):82–93. 10.1016/S0140-6736(04)17670-8. 15639683 10.1016/S0140-6736(04)17670-8

[19] Kunitoh H, Tsuboi M, Wakabayashi M, Okada M, Suzuki K, Watanabe S, et al. A phase III study of adjuvant chemotherapy in patients with completely resected, node-negative non-small cell lung cancer (JCOG 0707). JTCVS Open. 2020;4:90–102. 36004301 10.1016/j.xjon.2020.08.009 PMC9390442

[20] Shukuya T, Takamochi K, Sakurai H, Yoh K, Hishida T, Tsuboi M, et al. Efficacy of adjuvant chemotherapy with tegafur-uracil in patients with completely resected, node-negative NSCLC—real-world data in the era of molecularly targeted agents and immunotherapy. JTO Clin Res Rep. 2022;3(5):100320. 35601927 10.1016/j.jtocrr.2022.100320 PMC9117917

